# IFN Regulatory Factor 8 Is a Key Constitutive Determinant of the Morphological and Molecular Properties of Microglia in the CNS

**DOI:** 10.1371/journal.pone.0049851

**Published:** 2012-11-14

**Authors:** Carsten Minten, Rachael Terry, Celine Deffrasnes, Nicholas J. C. King, Iain L. Campbell

**Affiliations:** 1 The School of Molecular Bioscience, The University of Sydney, New South Wales, Australia; 2 The Discipline of Pathology, The University of Sydney, New South Wales, Australia; 3 The Bosch Institute, The University of Sydney, New South Wales, Australia; National Institute of Allergy and Infectious Diseases - Rocky Mountain Laboratories, United States of America

## Abstract

IFN regulatory factor (IRF) 8 is a transcription factor that has a key role in the cellular response to IFN-γ and is pivotal in myeloid cell differentiation. Whether IRF8 plays a role in the development and function of microglia, the tissue-resident myeloid cells of the brain, is unknown. Here, we show IRF8 is a constitutively produced nuclear factor in microglia, which suggested that IRF8 might also be a key homeostatic transcriptional determinant of the microglial cell phenotype. In support of this, in mice with a targeted disruption of the IRF8 gene, microglia were increased in number and showed gross alterations in morphology and surface area. *In situ* analysis of some key myeloid markers revealed that IRF8-deficient microglia had significantly reduced levels of Iba1, but increased levels of CD206 (mannose receptor) and F4/80 as well as increased tomato lectin binding. Analysis of microglia *ex vivo* revealed IRF8-deficient microglia had significantly increased levels of CD45, CD11b and F4/80, but significantly decreased levels of the chemokine receptors CCR2, CCR5 and CX3CR1. The known involvement of some of these molecular markers in membrane dynamics and phagocytosis led us to examine the phagocytic capacity of cultured IRF8-deficient microglia, however, this was found to be similar to wild type microglia. We conclude IRF8 is a constitutively produced nuclear factor in resident microglia of the CNS being a crucial transcriptional determinant of the phenotype of these cells in the healthy brain.

## Introduction

Microglia are myeloid lineage cells and the principal resident immune cells of the CNS (for reviews see [1], [2]). In the normal brain, these cells have a surveillant function but following perturbation of the local environment this can result in rapid transformation of these cells to highly active effector cells. Although much effort has been directed toward characterizing the fundamental properties of an activated microglial cell, surprisingly little is known about the intrinsic molecular mechanisms that program the functional state of these cells in either the healthy or the diseased CNS. Ultimately, the nature of the qualitative and quantitative alterations in gene transcriptional activity of microglial cells will determine largely which functional phenotype these cells assume. In a recent study aimed at resolving the transcriptional machinery that governs the microglial cell response to the cytokine interferon (IFN)-γ, we identified interferon regulatory factor (IRF) 8, as a prominent, constitutively expressed and IFN-γ-stimulated gene product in microglia [3].

IRF8, also termed IFN consensus sequence binding protein (ICSBP) is a member of the IRF family of transcription factors (reviewed in [4]). Members of this family are characterized by having a N-terminal DNA binding domain and a C-terminal IRF association domain (IAD), which is responsible for heterodimerization with other transcription factors. In general, the IRFs have key roles in IFN signalling pathways and hence are crucial in innate and adaptive immune responses [5]. While originally identified as a negative regulator, subsequent work indicated that IRF8 also stimulates the transcription of many genes [6], [7]. In particular, IRF8 has a key role in the cellular response to IFN-γ where it mediates a second wave of IFN-γ-driven gene transcription [6], [7]. The DNA binding activity of IRF8 alone is very weak, but dramatically increases through interaction with other transcription factors, particularly with other members of the IRF (e.g. IRF1, IRF2, IRF4) family and with members of the ETS (e.g. PU.1, TEL) family [6], [7]. Whether IRF-8 positively or negatively regulates transcription depends largely on which of these other transcription factors it interacts with.

IRF8 regulates cell growth and induces genes that promote macrophage and dendritic cell differentiation [4], [8]. Much of what we know about IRF8 has come from studies in IRF8-deficient mice derived from the targeted disruption of the *Irf8* gene [9]. These animals have increased numbers of granulocytes and macrophages in the spleen and lymph nodes while T-cell development and selection is apparently normal [9]. Aged IRF8-deficient mice develop a chronic myeloid leukemia-like disease [9]. IRF8-deficient mice also have increased susceptibility to infection with viral pathogens which is associated with impaired dendritic cell development and defective production of IL12p40 and consequently IFN-γ [9]. The extent to which IRF8 controls the myeloid cell response to signalling factors other than IFN-γ is less well studied. However, reconstitution of IRF8-deficient early myeloid progenitor cells with IRF8 in the presence of GM-CSF, results in the upregulation of a number of genes involved in macrophage differentiation [10]. Taken together these observations indicate IRF8 clearly has a more general role as transcriptional determinant of monocyte/macrophage function.

In light of the known aforementioned functions of IRF8 in monocyte/macrophage function together with our recent finding [3] that IRF8 is present in microglia, we hypothesised that this transcription factor may be a key intrinsic molecular determinant of the function of these cells in the healthy as well as the perturbed CNS. The major objective of the current study was to investigate this hypothesis in more detail and in particular, to determine if IRF8 is involved in microglial cell homeostasis in the healthy murine brain.

## Results

### IRF8 is a Constitutive and IFN-γ Stimulated Nuclear Factor in Microglia

**Figure 1:**
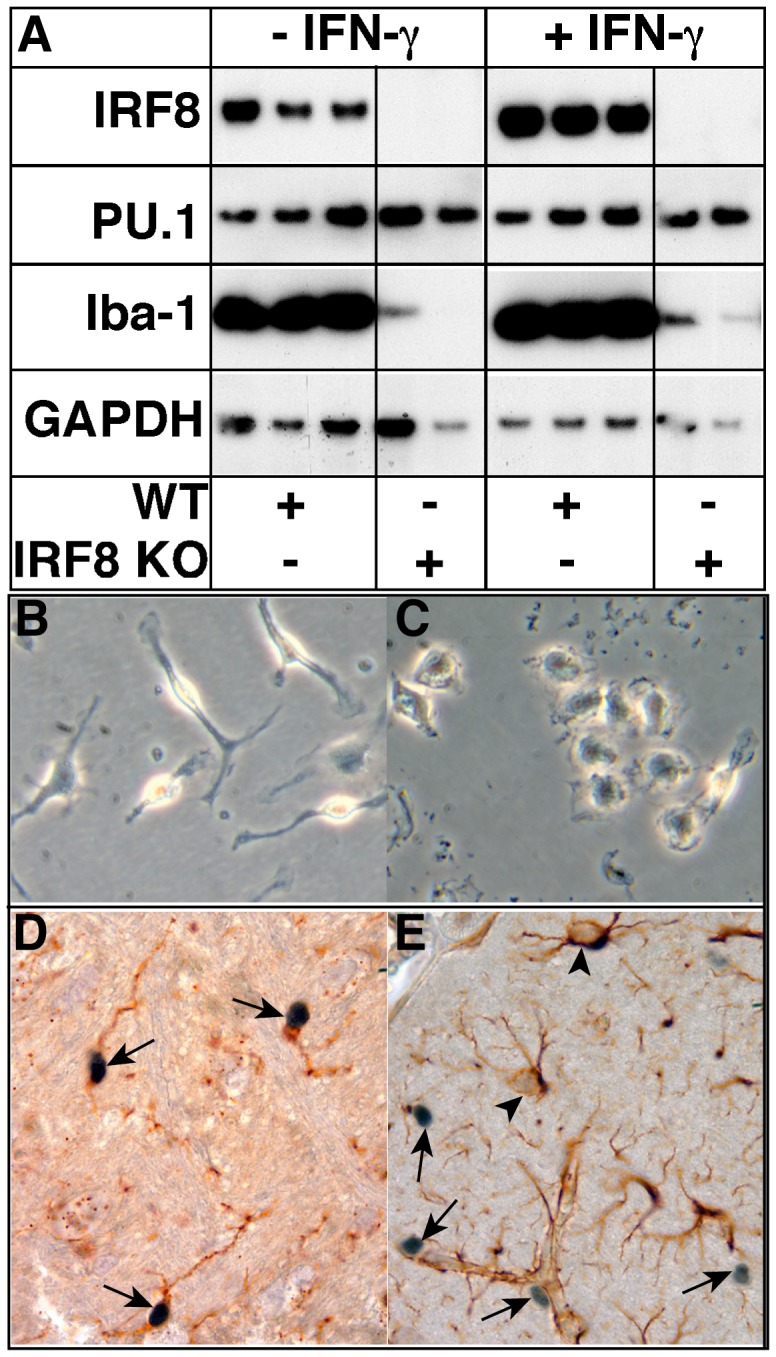
Comparative features of cultured microglia from WT versus IRF8-deficient mice and localization of IRF8 in the brain. Microglial cell cultures were prepared from the brain of neonatal mice as described in the Materials and Methods. Western blot analysis was performed on lysates of WT and IRF8-deficient primary microglia following treatment with (100 U/ml) or without IFN-γ for 4 h. GAPDH was used as a loading control (A). Morphological appearance of WT (B) or IRF8-deficient (C) microglia in primary culture (original magnification panels B&C, 400X). Immunohistochemical detection of IRF8 (D & E, arrows) combined with histochemical staing for tomato lectin binding (D) or GFAP immunohistochemistry (E, arrowheads). performed on brain sections from healthy adult mice. IRF8 staining is mostly confined to the nucleus of the cells (original magnification panel D, 1000X).

Previous experiments by us investigating the expression of various transcription factors involved in IFN-γ actions identified IRF8 as an IFN-γ-regulated gene in microglia [3]. Here we compared cultured neonatal WT as well as IRF8-deficient microglia following treatment with or without IFN-γ (Fig. 1A). Similar to our previous finding, IRF8 was found to be constitutively present in WT microglia and the levels increased following exposure to IFN-γ (Fig. 1A). By contrast, IRF8 was not detectable in microglia from IRF8-deficient mice treated with or without IFN-γ confirming the genotype of these animals and validating the specificity of the anti-IRF8 antibody used in our study. In comparison with IRF8, the ETS transcription factor PU.1 was present constitutively and at similar levels in both WT and IRF8-deficient microglia and the levels were not altered after treatment with IFN-γ. By microscopic examination, clear morphological differences were observed between neonatal WT (Fig. 1B) and IRF8-deficient (Fig. 1C) microglia in primary culture. While cultured microglia from WT mice appeared elongated with some branching processes, those from IRF8-deficient mice were amoeboid in shape and lacked branching processes.

We next asked whether IRF8 was present in microglia in the normal mouse brain. Double-labeling combining immunohistochemical detection of IRF8 with tomato lectin histochemistry was used to identify microglia (Fig. 1D). This analysis revealed numerous cells scattered throughout the brain parenchyma and associated with blood vessels that stained positive for IRF8 (Fig. 1D & 1E, arrows). Coincidently, all IRF8-positive cells stained positive for tomato lectin binding (Fig. 1D, arrows) but not GFAP (Fig. 1E, arrowheads) confirming that these cells were microglia. In addition, the IRF8 immunostain revealed that this protein was located predominantly in the nucleus of the microglia. It should be noted that although tomato lectin histochemistry also stained vascular endothelial cells, localization of IRF8 immunostaining to these cells was not observed. In summary, these findings indicate that IRF8 is a constitutively produced and IFN-γ-stimulated nuclear factor in microglia the absence of which does not alter the levels of another key myeloid transcription factor PU.1 but is associated with a significant alteration in the morphology of cultured microglia.

### IRF8-deficient Microglia In Situ Display Gross Changes in Morphology and Surface Area

The previous findings suggested that IRF8 may play a role in the homeostatic transcriptional regulation of microglia. To investigate this further, the impact of IRF8-deficiency on microglia in the brain was analyzed by confocal laser scanning microscopy. In order to visualize microglia within the murine brain we used transgenic (so-called MacGreen mice) mice that express eGFP under the control of the CSF1R (c-fms) promoter [11] resulting in eGFP-positive microglia within the brain. Either WT or IRF8-deficient mice were inter-bred with MacGreen mice to generate WT-MacGreen or IRF8-deficient-MacGreen mice, respectively. As shown by flow cytometry, analysis of microglia isolated directly from adult WT-MacGreen mice and from IRF8-deficient-MacGreen mice showed that the levels of eGFP were similar (see below). In addition, similar levels of eGFP were found to be expressed by splenic monocytes from WT-MacGreen and IRF8-deficient MacGreen mice (not shown) Thus, the absence of IRF8 did not alter the level of transcription of the transgene CSF-1R promoter confirming the utility of the MacGreen model for these studies.

**Figure 2:**
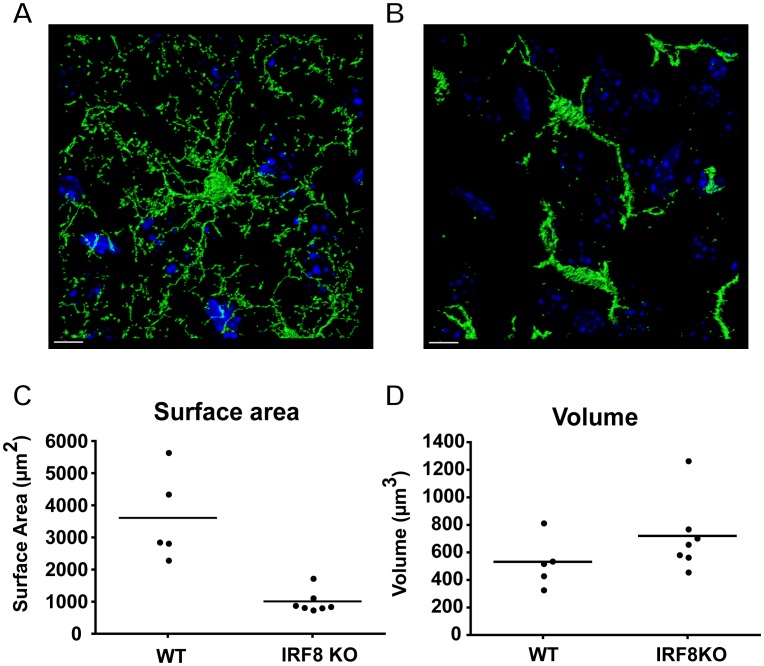
IRF8-deficient microglia have a marked reduction in ramification and surface area. Representative confocal images of eGFP positive WT (A) and IRF8-deficient (B) microglia in the cortex, counterstained with DAPI (blue) were obtained from 100 µm vibratome sections on a Zeiss LSM 510 Meta confocal microscope. The 3D analysis (surface rendering) was performed using Bitplane Imaris software. Size bar = 10 µm. Quantification of single-cell surface area (C) and volume (D) for microglia in the cortex of WT (n = 5) or IRF8-deficient (n = 7) mice. Values are shown with the bar representing the mean. IRF8-deficient microglia showed a significant (p<0.0025; Mann-Whitney U test) reduction in cell surface area and although cellular volume of these cells was increased slightly this was not significant.

Confocal image analysis of microglia in the cortex of WT-MacGreen mice, showed these cells were highly ramified and their elaborate fine processes appeared to form a network that extended throughout the volume of tissue analyzed (Fig. 2A). By contrast, in IRF8-deficient-MacGreen mice (Fig. 2B), the microglia were wider and shorter and lacked the fine processes characteristic of ramified microglia in the WT brain. Consequently, the brain volume occupied by the processes of IRF8 deficient microglia was considerably less than that seen in the WT-MacGreen brain. Therefore, as can be observed in Fig. 2B, areas of the nervous parenchyma were devoid of microglial processes. Quantifying the volume and cell surface area of individual cells within the confocal image stacks revealed that the cellular volume of IRF8-deficient-MacGreen microglia was slightly increased, but this did not reach statistical significance (Fig. 2D). However, consistent with their reduced morphological complexity, IRF8-deficient microglia had significantly less surface area being only one quarter of that of WT-MacGreen microglia (Fig. 2C). When investigating microglial numbers within a defined volume of brain tissue, we found that in comparable regions within the cerebral cortex and the cerebellum, IRF8-deficient mice had significantly more microglia (1.497±0.163 fold; p = 0.025) than WT mice.

Together, these findings highlight marked differences in the appearance and size of microglia in the brain of IRF8-deficient mice and suggest that IRF8 is a determinant of microglial morphology and number in the murine brain under homeostatic conditions.

### IRF8-deficient Microglia have Altered Molecular Properties

**Figure 3:**
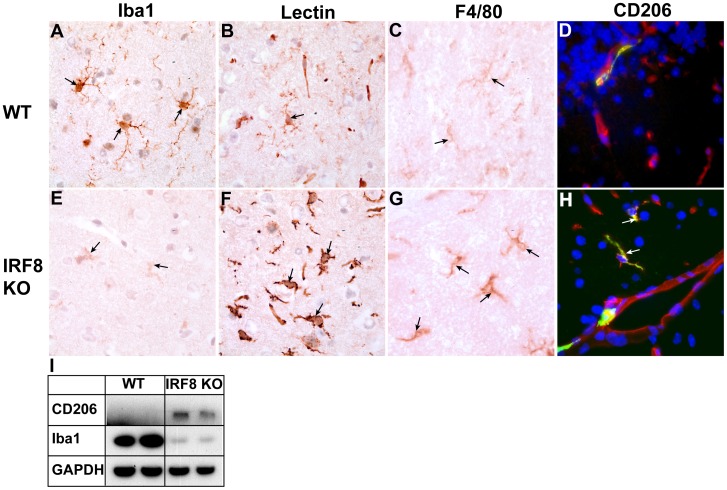
The levels of some key myeloid markers are altered in the brain of IRF8-deficient mice. Immunostaining was performed on brain sections from healthy, adult WT (A–D) or IRF8-deficient (E–H) mice as described in the Materials and Methods. Panels A, B, C, E, F, G show cortex while panels D, H show cerebellum (original magnification all panels 1000X). For immunofluorescence (G, H) DAPI was used to stain nuclei. Whole brain lysates were prepared from healthy, adult mice and 20 µg of protein analysed by western blotting (I).

We next analyzed the levels of some key myeloid and microglial cell markers *in situ* in WT versus IRF8-deficient mice. Notably, and compared with WT (Fig. 3A, arrows), there was a marked reduction in Iba1 staining of microglia throughout the brain of IRF8-deficient mice (Fig. 3E, arrows). Concordant with this finding, in IRF8-deficient mice steady-state levels of Iba1, as determined by immunoblotting, were found to be significantly reduced in primary cultured microglia (Fig. 1A) as well as in the brain (Fig. 3I). Converse to Iba1, microglia from IRF8-deficient mice showed higher levels of tomato lectin binding (Fig. 3F, arrows) and higher F4/80 expression (Fig. 3G, arrows) when compared with WT controls (Fig. 3B, arrows & 3C, arrows, respectively). Immunofluorescence staining for the mannose receptor (CD206) revealed CD206 expression was limited to lectin-positive cells within the perivascular space, the meningeal vessels and choroid plexus in WT brain (Fig. 3D). However, in the IRF8-deficient mice, CD206-positive microglia were dispersed throughout the brain parenchyma (Fig. 3H, arrows) which was reflected by increased steady-state levels of CD206 in the brain (Fig. 3I).

**Figure 4:**
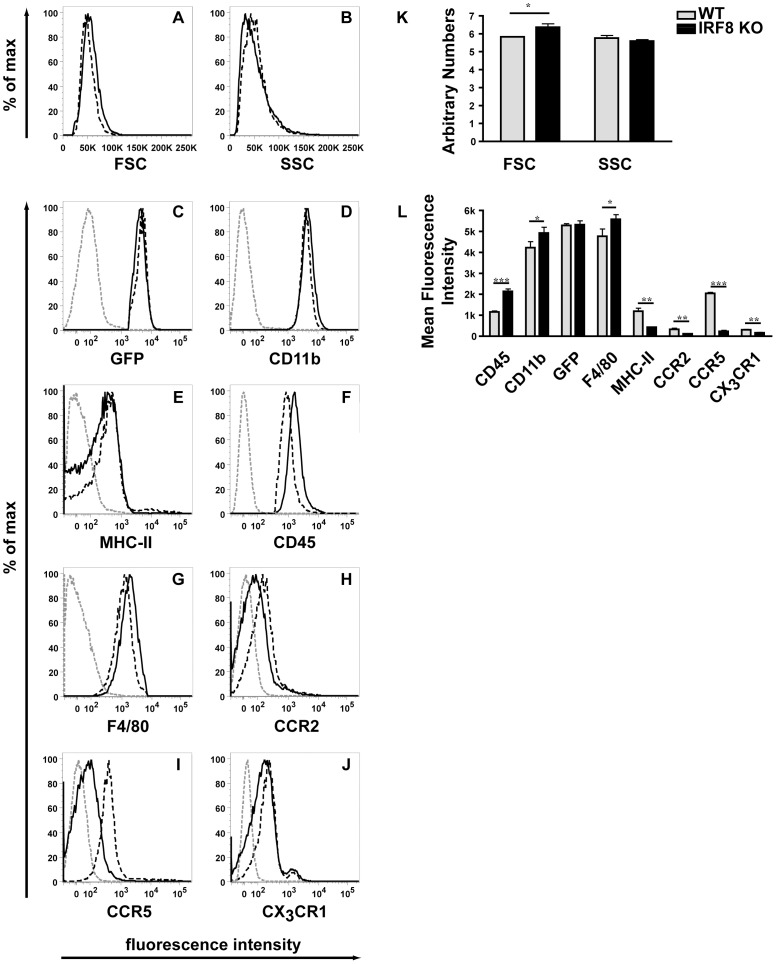
GFP^+^ cells in the CNS are exclusively CD11b^+^ microglia. Flow cytometry was performed on cells isolated from the brain of healthy adult WT (A, B) and IRF8-deficient mice as described in the Materials and Methods. For analysis, cells were scatter-gated to exclude dead cells and GFP^+^ cells were selected (A). Greater than 99% of GFP^+^ cells expressed CD11b in WT and IRF8-deficient mice (B), consistent with a microglial phenotype. Isotype matched antibodies were used to determine background staining (data not shown).

To further delineate their physical and molecular properties, microglia were isolated from the brain of healthy, adult WT-MacGreen or IRF8-deficient-MacGreen mice and analyzed by flow cytometry. We first assessed whether the GFP positive cell population in the brain was concordant between WT and IRF8-deficient mice (Fig. 4). This analysis revealed that of the gated GFP positive cells >99% were also positive for CD11b in brain from both WT and IRF8-deficient mice. This finding showed convincingly that the GFP positive cells correspond to microglia in the brain of MacGreen mice and verified that these populations of cells are comparable between the WT and IRF8-deficient mice.

**Figure 5:**
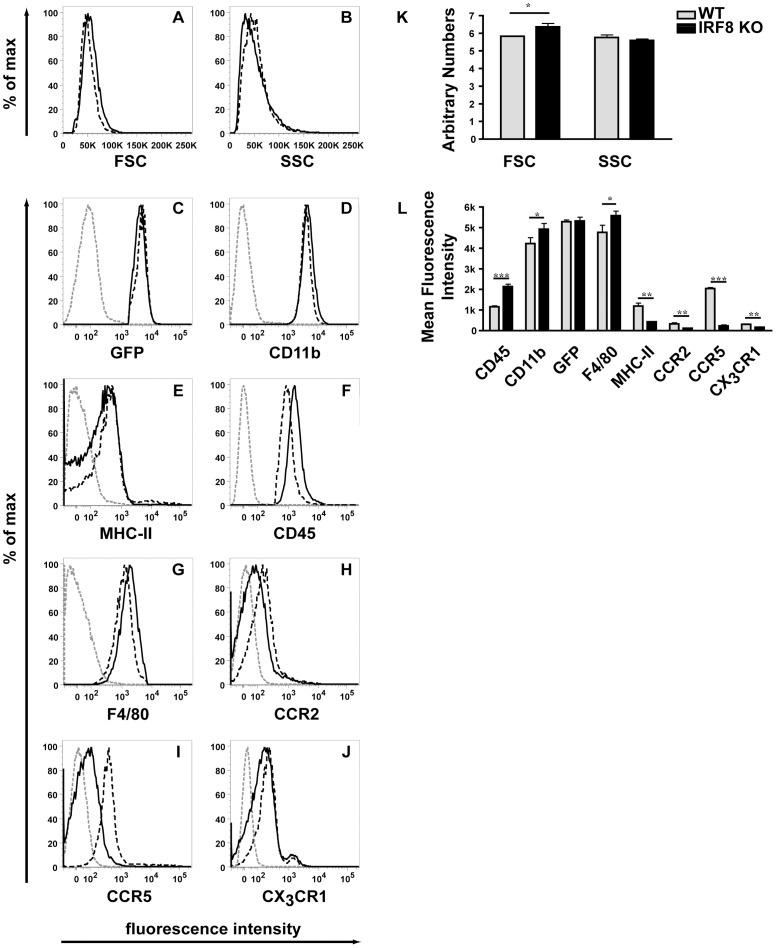
Surface levels of various molecules are altered on IRF8-deficient microglia. Flow cytometry (A-J) was performed on microglial cells isolated from the brain of adult WT (black dashed line) and IRF8-deficient (black line) mice as described in the Materials and Methods. For analysis, cells were scatter-gated to exclude dead cells and were selected for GFP, CD11b expression. An isotype matched antibody was used as a negative control (grey dashed line). Quantification of forward and sideward scatter; (K) or mean fluorescent intensity of histograms (L). The histograms represent means +/− SD from three separate experiments. For significance: ***P<0.001, **P<0.01, *P<0.05; by two-tailed t-test.

We next analysed the physical properties (Fig. 5A&B) and the levels of various surface markers (Fig. 5C–J) on the GFP positive microglia isolated from the brain of WT or IRF8-deficient mice. A small but significant increase was observed in the volume (FSC) but not the granularity (SSC) of IRF8-deficient microglia compared with WT microglia (Fig. 5K). The levels of various cell surface molecules including CD11b (Fig. 5D), CD45 (Fig. 5F), F4/80 (Fig. 5G) or MHC class II (Fig. 5E) and CX3CR1 showed a modest yet significant increase (Fig. 5L) or decrease (Fig. 5L), respectively in IRF8-deficient microglia compared with WT microglia. Similar changes were observed for WT and IRF8 KO microglia gated to select cells of the same FSC and SSC (data not shown), indicating that the modest changes observed were not due to differences in the volume of the cells. Finally, a more substantial and significant (Fig. 5L) decrease was observed in the levels of CCR2 (Fig. 5FH) and CCR5 (Fig. 5I) on IRF8-deficient microglia when compared with WT microglia. These findings indicated that IRF8-deficient microglia exhibit significant alterations in the expression of a variety of key molecular markers.

### IRF8-deficient Microglia Show No Defect in Phagocytosis

**Figure 6:**
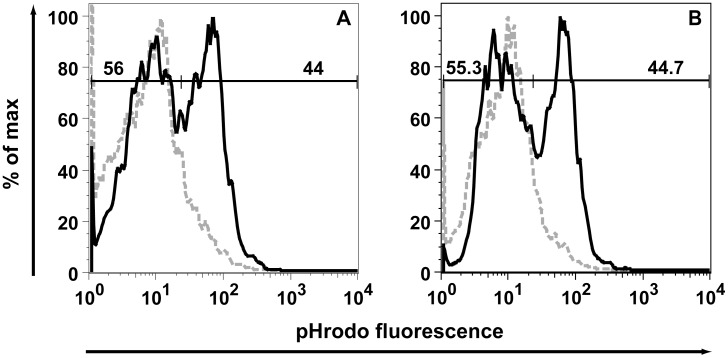
IRF8-deficient microglia show no defect in *E.coli* particle phagocytosis. Flow cytometry analysing pHrodo fluorescent *E.coli* particle uptake after 3 h by WT (A) and IRF8-deficient (B) primary microglia (grey dashed line = 4°C control assay, black continuous line = 37°C). Cells were gated for eGFP and CD11b (CD11b-PerCP-Cy5.5) and pHrodo fluorescent *E.coli* particle uptake was determined by measuring signal increase in the red fluorescence channel (fl2).

Some of the molecular markers that exhibited significant changes e.g. Iba1 [12] and the tomato lectin binding targeting poly-*N*-acetyl-lactosamine residues [13] in IRF8-deficient microglia noted in these experiments have been shown to be involved in membrane dynamics and phagocytosis which led us to examine the phagocytic capacity of IRF8-deficient microglia. The phagocytosis of fluorescently-labelled *E.coli* particles was assessed in primary cultured microglia isolated from the brain of WT or IRF8-deficient mice. While minimal phagocytosis was observed in control microglia at 4°C, increasing the temperature to 37°C resulted in a significant increase in the internalisation of the fluorescent particles (Fig. 6). However, no significant difference was observed in the amount of fluorescent particle ingestion between WT and IRF8-deficient microglia investigated at 3 h. Additional time points were analysed (20 min, 40 min, 60 min, 2 h), but no significant difference was found (data not shown). These findings suggest that IRF8-deficient microglia do not have a defect in phagocytosis.

## Discussion

Previously we observed in cultured microglia that not only was IRF8 an IFN-γ-stimulated factor but also was constitutively produced by these cells [3]. The present study has confirmed and extended these initial observations and found that IRF8 is constitutively produced in microglia in the CNS of mice and is located predominantly in the nucleus. While IRF8 was recently reported to be induced in cultured oligodendrocytes by IFN-γ [14], we could only detect IRF8 in microglia in the normal mouse brain suggesting that IRF8 is restricted largely to microglia *in vivo*. Whether IRF8 is inducible by IFN-γ in other cell types of the CNS *in vivo* remains to be determined.

Our finding that IRF8 is a constitutive nuclear factor in microglia also suggested that IRF8 may mediate actions in microglia that are independent of IFN-γ and is consistent with the known function of IRF8 to regulate myeloid cell development and function [9], [10]. IRF8 is a transcription factor, whose function depends on its interaction with other transcription factors including IRF1, IRF2 and the ETS factor PU.1 [6], [7]. Like IRF8, both IRF1 [3] and PU.1 [3], [15] are constitutively produced by microglia and, in the case of PU.1, is known to be located in the nucleus. Although IRF8 was reported to be a transcriptional repressor of PU.1 in B-cells [4], this was not evident in the current study where PU.1 levels remained unaltered in IRF8-deficient microglia. Studies in THP1 myelomonocytic leukemia cells with IRF8 siRNA knockdown [16] and murine macrophages deficient for IRF8 [17] identified a large number of genes that were regulated directly by IRF8 with a subset being dependent on PU.1 [16]. In light of these findings it is likely that IRF8 functions as a homeostatic transcriptional regulator in microglia.

Deficiency of IRF8 in mice causes myeloproliferative disease and immunodeficiency and affected animals are more susceptible to viral and bacterial infection [9], [18]. The central role of IRF8 in host defence and myeloid cell development is not restricted to mice and an inactivating mutation in the IRF8 gene in humans is associated with an autosomal recessive severe immunodeficiency with a complete lack of circulating monocytes and dendritic cells [19]. To date however, the impact of IRF8-deficiency on tissue-resident macrophages has not been reported. Significantly, our findings indicate that the tissue-resident macrophages of the CNS, the microglia are present in IRF8-deficient mice and indeed, are found in increased numbers. The basis for the increased numbers of resident microglia in the CNS of IRF8-deficient mice remains to be determined but likely reflects altered dynamics of these cells and could arise from increased local proliferation and/or longevity. Experimental evidence indicates that resident microglia are an ontogenically distinct population in the mononuclear phagocyte lineage and are derived from progenitors in the yolk sac that colonize the brain from around E9.5 [20], [21]. Our findings here suggest that IRF8 is likely not critical for this embryonic colonization by microglial progenitors and the subsequent migration and proliferation of these cells in the developing brain. However, cannot exclude a requirement for IRF8 for the differentiation of microglial progenitors into the characteristic ramified cell in the normal brain [22], [23].

A defining feature of resident microglia in the unperturbed brain is their distinct morphology consisting of a small cell body with extensive thin, long and elaborately branched processes. In the absence of IRF8 we showed that microglia were considerably less ramified and displayed rather stunted and swollen processes. Evidence suggests that the dynamics and elaborate arborization of microglial processes is dependent on a number of cellular and soluble cues in the microenvironment [24]. IRF8 may play an important role in programming the microglial response to these local cues or have a more general role in regulating the morphology of these cells. In support of the latter, IRF8-deficient microglia maintained an amoeboid morphology in culture that was quite distinct from WT microglia. The dramatic morphological change observed in IRF8-deficient microglia may also correspond to an alteration in the functional state of these cells. In response to nearly all brain perturbations such as damage or infection microglia undergo transformation to an active or effector state which is accompanied by distinct changes in morphology and molecular properties that are dependent on the nature of the insult and the signals involved [24], [25]. Morphologically, microglial activation usually involves a reduction in the degree of ramification with processes being more stunted and swollen and is accompanied by an increase in the volume of the soma. These changes are similar to the appearance of microglia in the brain of IRF8-deficient mice and suggest that these cells may be in a more activated state.

The number of microglia in the CNS varies by region being higher in gray matter versus white matter but is fairly evenly distributed within a given region [26]. The elaborate morphology and even distribution of microglia presumably allows the efficient surveillance of the entire tissue. However, in IRF8-deficient mice, surveillance may by compromised since the surface area of the cells is markedly reduced and, although offset by increased numbers of cells, areas of brain tissue were clearly visible around the microglia that were not invested with microglial processes. Other than surveillance for infectious agents and injured cells, the role of microglia in the healthy brain is less well understood. There is growing evidence that microglia contribute to normal brain physiology through interactions with neuronal and non-neuronal elements where they may be involved in fundamental processes such as phagocytosis of dying cells and synaptic remodelling [27]. However, we have not observed gross structural changes in the brain or overt behavioural abnormalities in IRF8-deficient mice, however, it cannot be ruled out that more subtle alterations exist that affect the CNS in these animals.

In addition to their unique morphology, resident microglia also possess a large number of molecular markers that define the myeloid lineage origins and functional properties of these cells. Here we surveyed a number of key myeloid and hematopoietic markers by IHC and flow cytometry and observed significant changes in the level of expression of a number of these markers by microglia from IRF8-deficient mice. Whether the changes observed occur as a result of the direct involvement of IRF8 in the regulation of the expression of the genes for these molecules or reflect indirect mechanisms such as altered responsiveness of the microglia to local cues is unclear presently and will require further investigation. However, a review of the genes reported to be significantly regulated by IRF8 in resting BMDMs [17] revealed that the *Aif1* (allograft inflammatory factor 1 gene [28] (which corresponds to Iba1 [29]), is strongly upregulated by IRF8. Thus, loss of IRF8 stimulation likely accounts for the significant reduction in Iba1 expression we have observed in IRF8-deficient microglia.

The expression of Iba1 is restricted to the monocytic lineage and has been used widely to discriminate microglia in the CNS [24], [30]. The levels of Iba1 are increased in activated microglia [31] where it is a key molecule involved in plasma membrane dynamics [12]. It is therefore possible that the alterations in microglial morphology found with IRF8-deficiency may be due in part, to the reduction in Iba1 and a consequent change in the properties of the plasma membrane.

In addition to Iba1, other molecules that were decreased significantly in IRF8-deficient microglia were the chemokine receptors CCR2, CCR5 and CX3CR1. There are no reports of a link between IRF8 and the regulation of the genes for these chemokine receptors. However, in other studies we have observed that CCR2 and CCR5 are also decreased on circulating monocytes from IRF8-deficient mice (Terry, Minten, Campbell and King: unpublished observations) suggesting that the decrease in these receptors represents a more general response to the absence of IRF8. CCR2 and CCR5 bind a number of key inflammatory chemokines including CCL2 (CCR2) and CCL1, CCL4 and CCL5 (CCR5). These chemokines are known to be induced in the CNS in association with a variety of neuroinflammatory states where they function chiefly to modulate cell migration [32]. The significant decrease in the levels of the key receptors for these cytokines suggests that the response of IRF8-deficient microglia to these chemokines may be compromised. On the other hand, CX3CR1 is the receptor for CX3CL1, a chemokine that is produced constitutively in the CNS by neurons and may facilitate communication between neurons and microglia [33]. Disruption of this dialogue between neurons and microglia may result in altered microglial and neuronal function. Thus, mice with a genetic ablation of CX3CL1 exhibited enhanced microglial activation and neutotoxicity following toxin-induced inflammation and in genetic models of neurodegeneration [34].

In contrast to the decreased Iba1 and chemokine receptor levels, microglia deficient in IRF8 showed a modest but significant increase in a number of markers including CD45, CD11b and F4/80 as well as in tomato lectin binding. These molecules which have been used widely as targets to identify microglia are all increased with microglial activation [24], [30]. Together with the striking morphological changes noted above, the increased level of CD45, CD11b, F4/80 and tomato lectin binding might indicate an increased state of activation of the microglia in the IRF8 brain. The increased expression of the mannose receptor (CD206) we observed on IRF8-deficient microglia would also be consistent with this possibility. Although CD206 is limited to myeloid cells within the perivascular space, the meningeal compartment and in choroid plexus macrophages in the healthy brain [35], we found that CD206 was more widely distributed and included resident microglia in IRF8-deficient animals.

Among the genes that are positively regulated by IRF8 are genes related to lysosomal/endosomal enzymes crucial in macrophage phagocytic function [36]. In the present study gross changes were found in the levels of the Iba1 and CD206 molecules as well as in tomato lectin binding in IRF8-deficient microglia. These molecular entities are all involved with phagocytic function [12], [13], [37] which led us to investigate whether phagocytosis was altered in microglia that lacked IRF8. However, the findings clearly showed that the absence of IRF8 from microglia did not influence the capacity of these cells to engulf *E. coli* particles suggesting that phagocytic function of these cells remains intact.

In conclusion, our studies have identified IRF8 as a novel, constitutively produced nuclear factor in a tissue resident macrophage population, the parenchymal microglia of the CNS, and suggest that IRF8 is a crucial transcriptional determinant of microglial cell properties and function in the homeostatic state. Ongoing studies should provide further insight as to the explicit functions regulated by IRF8 in microglia of the healthy CNS. Moreover, given the central role of microglia in neuroinflammatory and neurodegenerative disease states [25], [38] and the recent identification of the *IRF8* gene as a susceptibility loci in MS [39], [40], it will be of considerable interest to also determine the impact of IRF8 on the function of these cells in the diseased CNS.

## Materials and Methods

### Animals

*Irf8* gene targeted knockout mice (C57BL6 background) described previously [9] were kindly provided by Dr. Stephen Nutt (Walter and Eliza Hall Institute of Medical Research, Melbourne, Australia). Some IRF8-deficient mice or wild type (WT) (C57BL6) mice were inter-crossed with CSF-1R-eGFP (MacGreen) transgenic mice ( [11]; obtained from the Transgenic Animal Service of Queensland, St. Lucia, Australia) to generate mice with eGFP-positive, WT microglia or eGFP-positive, IRF8-deficient microglia. All mice were kept under constant circadian rhythm, constant temperature and provided with food and water *ad libitum* and housed in filter top cages in the animal facility of the School of Molecular Bioscience at the University of Sydney. Ethical approval for the use of all mice in this study was obtained from the University of Sydney Animal Care and Ethics Committee.

### Isolation and Culture of Primary Microglia

Primary microglia were isolated as described previously [3]. Briefly, 2–4 day old mice were euthanized and brains removed, washed in PBS and cerebral cortices were separated, broken up into small pieces and digested in papain solution (1 mg/ml papain (Worthington, Lakewood, NJ, USA), 240 µg/ml L-cysteine (Sigma-Aldrich), 1140U DNase I type IV (Sigma-Aldrich), 25 mM HEPES (Invitrogen, Mulgrave, VIC) for one hour at 37°C. Tissue was homogenized, centrifuged (120 x g for 5 min.) and the cell pellet resuspended and plated onto poly-D-lysine-coated culture flasks. Mixed glial cells were split 1∶6 after 6 days in culture and 8–10 days later primary microglia were isolated by shaking. The microglia were then cultured separately in T25 flasks (BD Falcon) in DMEM containing 10% FBS. Prior to treatment, microglial cultures were incubated at 37°C for 12 h in the absence of serum followed by the addition of murine recombinant IFN-γ (Sigma-Aldrich; 100 U/ml) in DMEM for 4 h.

### Immunoblotting

Following treatment as described above, cultured microglial cells were washed in PBS (x2) and then solubilized in Tris-buffered (50 mM, pH 7.5) lysis buffer containing Nonidet-P40 (Sigma-Aldrich; 1.5% v/v), protease inhibitor cocktail (1∶50 v/v, Calbiochem, Kilsyth, VIC), phosphatase-inhibitor cocktail (1∶50 v/v, Calbiochem), 1 mM EDTA, 1 mM DTT and 10% (v/v) glycerol. Samples were clarified by centrifugation (10000 x g for 10 min) at RT and the protein concentration was determined by bicinchoninic assay (BCA) (Pierce, Rockford, IL, USA). Samples (5 µg of protein) were resolved on a 4–12% sodium dodecylsulfate polyacrylamide gel (NuPAGE, Invitrogen) and electroblotted onto a polyvinylidene difluoride membrane (PVDF, GE Healthcare). Membranes were blocked and incubated overnight at 4°C in primary antibody against: IRF8 (diluted 1∶1000, Santa Cruz Biotechnology, Santa Cruz, CA, USA); Iba1 (diluted 1∶1000, Wako Chemicals, Richmond, VA USA); GAPDH (diluted 1∶10000, Millipore, North Ryde, NSW); PU.1 (diluted 1∶1000, Cell Signalling Technology, Danvers, MA, USA), washed and incubated for one hour with HRP-conjugated secondary antibody (anti-rabbit-HRP and anti-goat-HRP both from Santa Cruz; anti-mouse-POX from Sigma-Aldrich) at RT. Specific protein bands were visualized using chemiluminescent HRP-substrate (Millipore) and X-ray film (Amersham Hyperfilm ECL, GE Healthcare).

### Microglial Cell Isolation and Ex Vivo Analysis by Flow Cytometry

Mice were anesthetized and perfused intra-cardially using 20 ml ice-cold PBS. Brains were dissected and processed as described previously [41]. Briefly, brains were passed through a coarse metal sieve and digested in collagenase IV (Sigma-Aldrich, Castle Hill, NSW) and DNase I (Sigma-Aldrich) for 1 h at 37°C. The reaction was stopped by adding 10% FCS, non-digested tissue was removed as samples were passed through a 70 µm sieve, and centrifuged for 15 min at 340×g. The cell pellet was then suspended in 30% Percoll (GE Healthcare, Castle Hill, NSW) and layered over 80% Percoll. Samples were centrifuged at 1140×g for 25 min at room temperature, and cells at the interface were collected for further analysis.

Isolated cells were washed in PBS and blocked with anti-CD16/CD32 antibody (Biolegend, San Diego, CA, USA), for 20 min. Viable cells were counted using trypan blue exclusion (Sigma-Aldrich), which routinely showed >95% cell viability. Cells were again washed in PBS and resuspended in the appropriate antibody cocktail for 1 hour. Fluorochrome-conjugated anti-CD45, CD11b, Ly6C, Ly6G, CD11c, MHC-II, CCR5 and F4/80 antibodies were obtained from Biolegend. The anti-CCR2 antibody was obtained from R&D Systems (Minneapolis, MN, USA) and the and CX_3_CR1 antibody was from Abcam (Cambridge, MA, USA). Cells were then washed and fixed in fixation buffer (Biolegend) for 20 min. Samples were then run on the FACS LSR-II (BD Biosciences, North Ryde, NSW) with acquired data files analyzed using the program FlowJo (Tree Star, Stanford, CA, USA). Quantification of cell populations of interest was calculated using percentages obtained from flow cytometry analysis and live cell counts.

### Tissue Processing and Immunohistochemistry

Mice were anaesthetized and perfused intra-cardially with 20 ml ice-cold sterile PBS. Brains were removed immediately and bisected down the sagittal midline. The hemi-brains were placed in PBS-buffered 4% paraformaldehyde overnight at 4°C. One hemi-brain was processed for paraffin embedding while the other hemi-brain was placed in 30% (v/v) sucrose for 24 h and then embedded in Tissue Tek OCT compound (Sakura Finetek, Alphen, The Netherlands) and frozen in a bath of dry-ice cooled 3-methylbutane (Sigma-Aldrich). Additional freshly bisected hemi-brains were immediately embedded in Tissue Tek OCT compound and flash frozen as described above. Five µm thick sections were obtained from paraffin-embedded tissue, deparaffinized in xylene and rehydrated through a series of graded ethanol solutions. Cryosections (8–20 µm) were obtained from fixed and unfixed tissue embedded in OCT compound, cut on a Leica CM1850 cryostat (Leica Microsystems, North Ryde, Australia). Sections were placed on slides, air-dried and stored at −20°C until use. Prior to immunohistochemistry cryostat sections were equilibrated to RT for 30 min in the presence of silica beads (Ajax Finechem, Taren Point, NSW).

The PFA-fixed tissue sections were processed prior to staining by treating the slides with either Tris-HCL buffer (25 mM, pH 8.5) containing 0.05% SDS (w/v) and EDTA (1 mM) for 40 min at 97°C or with sodium citrate buffer (10 mM, pH8.5) for 2 h at 50°C. Tissue sections treated with Tris-HCL buffer were then incubated overnight at 4°C with anti-Iba1 antibody (Wako Chemicals) while sections treated with sodium citrate buffer were incubated with CD206 (R&D Systems). Additional sections treated with sodium citrate buffer were also incubated for 1 h at RT with biotinylated tomato lectin (Sigma-Aldrich). After incubation with biotinylated secondary antibodies (diluted 1∶200, 1 h at RT; Vector Laboratories) followed by horseradish peroxidase (HRP)-coupled streptavidin (1∶200, 1 h at RT; Vector Laboratories). For lectin histochemistry, sections were treated with HRP-coupled streptavidin (1∶200, 1 h at RT; Vector Laboratories). In both cases, to visualize the final reaction product, a NovaRed Substrate kit (Vector Laboratories) was used according to the instructions recommended by the manufacturer. Finally, sections were counterstained with haematoxylin (Sigma-Aldrich) for 2 min. For dual-label staining (IRF8 and tomato lectin), sections were deparaffinized, rehydrated and treated with 10 mM sodium citrate (pH 6.0; 95°C for 15 min; Sigma-Aldrich) for antigen unmasking. Sections were incubated with goat-anti-IRF8 antibody (1∶200; Santa Cruz) overnight at 4°C. After washing, sections were incubated with biotinylated secondary antibody for 30 min and with streptavidin-HRP for 30 min. Visualization of IRF8 immunohistochemistry was performed using diaminobenzidine peroxidase substrate containing nickel (Vector Laboratories). Subsequently, sections were washed in distilled water and incubated for 15 min with avidin followed by biotin. For double-labelling with tomato lectin, sections were incubated with biotinylated tomato lectin for 1 h at RT. After washing, sections were incubated with streptavidin-HRP for 30 min and lectin binding visualized with NovaRed Substrate kit (Vector Laboratories). For controls, sections were stained in parallel as described above except the primary antibody was replaced with the equivalent dilution of non-immune sera of the appropriate species. In addition, for the IRF8 antibody immunostains were performed using sections from IRF8-deficient mice.

### Confocal Laser Scanning Microscopy and Image Analysis

Confocal microscopy was performed on a Zeiss 510 Meta confocal microscope located at the Bosch Institute, University of Sydney. Sections (20 µm) were prepared as described above using a cryomicrotome. In addition, mice were perfused intra-cardially with 20 ml ice-cold sterile PBS and brains were fixed in 4% PFA overnight. Brain tissue was mounted in 4% high gel strength agar (Sigma-Aldrich) and 80 um thick sections cut on a vibratome (Leica Microsystems). Sections were mounted in 50% glycerol in 0.1 M phosphate buffer on slide and analysed with the confocal microscope. In brief, confocal images focussing though the entire tissue section were acquired. From these image stacks, three-dimensional images were produced using Imaris software (Bitplane AG, Zürich, Switzerland). Using the surface function of Imaris, cells were surface rendered and individual cells were chosen within one 80 µm deep 140×140 µm section and parameters such as volume and surface area calculated (WT: n = 5; IRF8-deficient: n = 7). Microglial cell number per volume was determined by counting eGFP positive cells manually within the acquired z-stacks using the Imaris software. In total, three z-stacks of each WT and IRF8-deficient brain tissue, two of which acquired were from 20 µm and one from 80 µm thick sections were used to determine microglial numbers.

### Phagocytosis

Microglial phagocytosis was determined by cellular uptake of pH-sensitive rhodamine-conjugated *E. coli* particles (pHrodo E.coli bioparticles, Invitrogen). Phagocytosis experiments were performed according to the manufacturer’s instructions. Briefly, 10^5^ primary microglial cells per well in DMEM plus 10% FBS were grown overnight in a 96-well plate. Microglial cells were washed once and culture medium was replaced with 100 µl E. coli particles in Hanks balanced salt solution (HBSS, Invitrogen) containing 20 mM HEPES (Invitrogen). The cells were incubated for various times at 37°C and a control cell preparation was incubated at 4°C. Cells were harvested, washed twice in PBS (supplemented with 1% FBS v/v) and analyzed by flow cytometry. Fluorescence from ingested *E. coli* particles was detected in fluorescence channel 2 (FL2) on a FACScalibur (BD Biosciences).

### Statistical Analysis

Statistical analysis was performed using either Prism V4 or InStat V3 (both from GraphPad, San Diego, CA, USA). Depending on the data to be analysed either a two-tailed t-test or Mann-Whitney U test were performed, with p≤0.05 considered significant.
