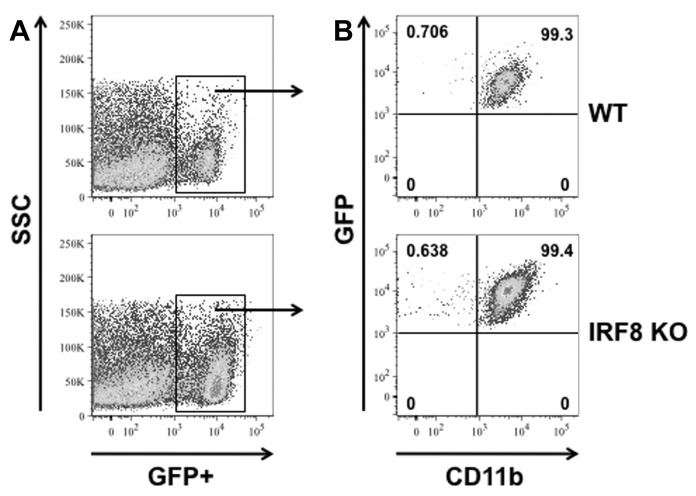# Correction: IFN Regulatory Factor 8 Is a Key Constitutive Determinant of the Morphological and Molecular Properties of Microglia in the CNS

**DOI:** 10.1371/annotation/492fdf80-c999-4947-b569-96af8cb4e9d9

**Published:** 2013-05-03

**Authors:** Carsten Minten, Rachael Terry, Celine Deffrasnes, Nicholas J. C. King, Iain L. Campbell

The version of Figure 4 included in the article is a duplicate of Figure 5. Please see the correct Figure 4 file here: 

**Figure:**